# Differences in alveolo-capillary equilibration in healthy subjects on facing O_2_ demand

**DOI:** 10.1038/s41598-019-52679-4

**Published:** 2019-11-13

**Authors:** Egidio Beretta, Gabriele Simone Grasso, Greta Forcaia, Giulio Sancini, Giuseppe Miserocchi

**Affiliations:** Dipartimento di Medicina e Chirurgia, Ambulatorio di Fisiologia Clinica e dello Sport, Scuola di Specializzazione in Medicina dello Sport, Università di Milano-Bicocca, Via Cadore, 48, 20900 Monza, Italy

**Keywords:** Respiration, Medical research

## Abstract

Oxygen diffusion across the air-blood barrier in the lung is commensurate with metabolic needs and ideally allows full equilibration between alveolar and blood partial oxygen pressures. We estimated the alveolo-capillary O_2_ equilibration in 18 healthy subjects at sea level at rest and after exposure to increased O_2_ demand, including work at sea level and on hypobaric hypoxia exposure at 3840 m (*P*_*A*_ ~ 50 mmHg). For each subject we estimated O_2_ diffusion capacity (*DO*_*2*_), pulmonary capillary blood volume (*Vc*) and cardiac output ($\dot{Q}$). We derived blood capillary transit time ${\boldsymbol{(}}{\boldsymbol{T}}{\boldsymbol{t}}{\boldsymbol{=}}\frac{{\boldsymbol{V}}{\boldsymbol{c}}}{\dot{{\boldsymbol{Q}}}}{\boldsymbol{)}}$ and the time constant of the equilibration process (${\boldsymbol{\tau }}{\boldsymbol{=}}\frac{{\boldsymbol{\beta }}{\boldsymbol{V}}{\boldsymbol{c}}}{{\boldsymbol{D}}{{\boldsymbol{O}}}_{{\boldsymbol{2}}}}$, *β* being the slope of the hemoglobin dissociation curve). O_2_ equilibration at the arterial end of the pulmonary capillary was defined as ${{\bf{L}}}_{{\bf{e}}{\bf{q}}}{\boldsymbol{=}}{{\bf{e}}}^{{\boldsymbol{-}}\frac{{\bf{T}}t}{{\boldsymbol{\tau }}}}$. *L*_*eq*_ greately differed among subjects in the most demanding O_2_ condition (work in hypoxia): lack of full equilibration was found to range from 5 to 42% of the alveolo-capillary *PO*_*2*_ gradient at the venous end. The present analysis proves to be sensible enough to highlight inter-individual differences in alveolo-capillary equilibration among healthy subjects.

## Introduction

The interaction between diffusion and perfusion in determining oxygen flow across the air-blood barrier is a fundamental factor sustaining metabolic oxygen need. Further, an efficient O_2_ diffusion-transport implies perfect equilibration for O_2_ partial pressure between the alveolar compartment and the blood flowing in the pulmonary capillary, that, in turn, allows to perfuse tissues with an optimal arterialization. The importance of this point becomes relevant when balanced against an increase in cells metabolic requirement, namely the increased oxygen demand, as in exercise, as well as in conditions causing a limitation to oxygen delivery (environmental hypoxia and cardio-pulmonary disorders). Recent work from our group revealed considerable inter-individual differences in the adaptive response of the air-blood barrier to work in normoxia and hypoxia^1–3^ and further the adaptations correlated with the morpho-functional phenotype of the air-blood barrier^4^.

Piiper and Scheid^5^ proposed an analysis to define the efficiency of the functional coupling for diffusion of oxygen with its blood transport capacity. This approach was strongly grounded on the principle of mass conservation whereby, under steady state conditions, the mass transfer of diffused O_2_ equals that received by the mixed venous blood affluent to the lung which, in turn, corresponds to the oxygen consumed by the tissues^6^. In this study we further developed the original model of Piiper and Scheid^5^ aiming at evaluating inter-individual differences in the efficiency of the lung O_2_ diffusion-perfusion mechanism among healthy subjects. To this aim, we defined the time constant of the O_2_ alveolo-capillary equilibration (*τ*), resulting from the complex interaction between the factors involved in the gas diffusion/transport function, namely: pulmonary capillary blood transit time (*Tt*), the overall lung diffusive capacity for O_2_ (*DO*_*2*_), cardiac output ($\dot{Q}$), and lung capillary blood volume (*Vc*). This approach led us to highlight remarkable differences among subjects in the O_2_ equilibration and to discuss the reasons for diffusion limitation.

## Materials and Methods

The research project was approved by the ethical committee of University of Milano Bicocca and was conducted in accordance with the Helsinki Declaration for research on humans to assure ethical standards were met. Participants were instructed about the experimental procedure and related discomfort, as well as of the risks of acute exposure to hypoxia. All individual participants included in the study received and signed an informed written consent.

### Participants

Data were obtained from 18 healthy participants (13 males, 5 females), average age 36.4 ± 8.2 (mean ± SD), and regularly practicing mountaineering and/or mountain hiking. All participants were no smokers or mild smokers (5 participants, smoking less than 4 cigarettes/day) and their spirometric parameters were above 90% of predicted values. Smokers were instructed to refrain from smoking 24 h before the test. All measurements were performed at sea level (SL, Monza Italy, 170 m, *P*_*I*_*O*_*2*_ 157 mmHg) and at high altitude (HA, Aiguille du Midi, 3840 m, *P*_*I*_*O*_*2*_ 90 mmHg), at rest and during work. Participants reached the laboratory at HA by cable car (about 30 minutes) and measurements were done after 4–6 hours from reaching the laboratory at HA. The four conditions considered in the study were defined as: SLrest, SLwork, HArest and HAwork.

### Exercise protocol

#### Incremental test

At sea level (SL) an incremental exercise test was performed on a cycloergometer^1^ (Ergoline 900, Cosmed, Italy). After 2 min of unloading pedaling, the workload was increased every 60 s until voluntary exhaustion. The initial workload at SL was set at 60–90 W (according to an estimated level of physical fitness) and increments of 15–25 W/min were given, up to exhaustion. The gas exchange threshold (*GET*) was determined as the point where the slope of the $\dot{V}{O}_{2}$ *vs* $\dot{V}E$ relationship increased^7^ and the corresponding heart rate was determined.

#### Constant workload test

To ensure a correct measurement of lung diffusive capacity for carbon monoxide (*DLCO*) and its subcomponents during exercise (*see below*), all participants performed a steady-state test lasting no less than 18 minutes. For each subject, the test was performed at a workload of 70% (at SL) and 60% (at HA) of that corresponding to the *GET* determined at SL. This condition assured that the oxygen flow was constant and equal along the transport chain^6^.

For each participant, at rest and during exercise we determined with a portable metabolic cart (K4b2, Cosmed, Roma, Italy): pulmonary ventilation ($\dot{V}E$, in BTPS), end tidal O_2_ and CO_2_ partial pressure (*PetO*_*2*_, *PetCO*_*2*_). Heart rate (*HR*) was continuously monitored and determined with a 12-lead electrocardiographic interfaced to the metabolic cart. Arterial blood O_2_ saturation (%*SatO*_*2*_) was monitored continuously through oximetry at the finger (RAD 9 Signal Extraction Pulse Oximeter: Masimo Corporation, Irvine - California, USA)^1^.

### Diffusion measurement

We relied on the Roughton and Forster^8^ model, according to the equation:

1 $$\frac{1}{DLCO}=\frac{1}{Dm}+\frac{1}{\theta \cdot (Hb)\cdot Vc}$$

where *DLCO* is the overall diffusion capacity of the lung, *Dm* and *Vc* are the diffusion subcomponent representing alveolar membrane diffusive capacity and lung capillary blood volume, respectively; *θ* is the binding rate of Hb with CO, (Hb) is the ratio between individual hemoglobin concentrations over reference values of hemoglobin concentration for men and women^9^.

Measurement of *DLCO* and subcomponents were performed according to standardized procedures^9^, in sitting position at total lung capacity (*TLC*) by single breath method (QUARK PFT, Cosmed, Roma, Italy), at SLrest and HArest and at a workload at *GET* (SLwork and HAwork). Participants inspired 3 gas mixtures containing 0.3% CH_4_ (tracer to measure alveolar lung volume, *VA*), 0.3% CO and 20, 40 and 60% O_2_, respectively. Each maneuver was performed at least 6 min after the previous one. For determination of *DLCO* during work, the subject stopped pedaling, on the average for less than 1 minute, at 6, 12 and 18 minutes to allow measurements to be done using 20, 40 and 60% O_2_, at the three time points, respectively.

*DLCO* values measured at HA were adjusted according to a recently developed method that accounts for the inter-individual differences of the effect of hypoxia exposure on diffusion subcomponents^2^, an approach allowing to maintain the validity of Eq. 1 on numerical basis. Blood samples were taken once to determine Hb concentration and haematocrit.

CO binding reflects individual Hb concentration, yet aiming to consider only the inter-individual differences in *DLCO* as reflecting the diffusive properties of the alveolar membrane, we standardized Hb concentration to reference values of 14.6 g/dl in men and 13.4 g/dl in women^9^. We relied on *θ* values from Forster^10^ according to the relationship 1/*θ* = 0.75 + (0.0057 · *P*_*A*_), where *P*_*A*_ is alveolar O_2_ partial pressure assumed equal to the measured end tidal O_2_ pressure.

We derived the *DO*_*2*_ values from measured *DLCO* multiplied by the constant 1.23^11^; this procedure was validated by measurement based on mass spectrometry using oxygen isotopes^11^, as well as with those calculated by Hammond and Hempleman^12^ and Hempleman and Gray^13^ based on *MIGET* (Multiple Inert Gas Elimination Technique).

### Echocardiography and cardiac output

As in previous study from our group^3^, standard 2D echocardiography was performed at rest in supine position using a portable echo machine with a 2.5–3.5 MHz cardiac probe (Vivid I, General Electric Healthcare Clinical System) by a single experienced cardiologist, both at SL and HA. Care was taken to ensure that the position of the participants and the transducer were similar in all examinations. Stroke volume was obtained from apical 4 chamber view. Cardiac output ($\dot{Q}$) was measured multiplying Left Ventricle outflow tract time-velocity integral, measured using pulse wave Doppler, by its cross-sectional area and heart rate^14^. To derive $\dot{Q}$ during exercise we assumed an increase in stroke volume of 40%, as from data provided by Poliner^15^ and Stöhr^16^.

### Estimate of O_2_ equilibration

We define the limitation of O_2_ equilibration at the exit of pulmonary capillary as *L*_*eq*_ (*see Appendix*):

2 $${L}_{eq}={e}^{-\frac{D{O}_{2}}{\beta \dot{Q}}}$$

where *DO*_*2*_ is the oxygen diffusive capacity, $\dot{Q}$ is the cardiac output and *β* is the hemoglobin binding capacity for oxygen, expressing the mean slope of the Hb-O_2_ dissociation curve (*see Appendix*).

Knowing blood transit time in the pulmonary capillaries (*Tt*) and the time constant of the equilibration process (*τ*), one can express diffusion limitation also as:

3 $${L}_{eq}={e}^{-\frac{Tt}{\tau }}$$

that allows to draw the exponential profile of the alveolo-capillary O_2_ equilibration as a function of time.

## Results

### Correlation between diffusive and perfusive variables involved in O_2_ equilibration

Table 1 reports means (±SD) and median (IQR 25–75) values of %*SatO*_*2*_ and *P*_*A*_ in the four conditions studied.

**Table 1:** Means (±SD) and medians (IQR 25–75) values of hemoglobin O_2_ saturation (%*SatO*_*2*_) and alveolar oxygen partial pressure (*P*_*A*_) in the four studied conditions.

		SLrest	SLwork	HArest	HAwork
%*SatO*_*2*_	*mean*	99 ± 1	98 ± 1**	88 ± 4	81 ± 6**
	*Median* (*IQR 25*–*75*)	99 (98–100)	98 (97–99)	88.5 (86–90)	83 (78–85)
*P*_*A*_, *mmHg*	*mean*	104 ± 2.7	103 ± 10.0	52 ± 2.3	55 ± 3.8*
	*Median* (*IQR 25*–*75*)	104.8 (102.6–105.0)	106.7 (94.7–109.7)	52.2 (50.7–53.3)	56.3 (52.2–56.8)

*Work vs rest p < 0.05.

**Work vs rest p < 0.01.

No significant differences were found for hemoglobin concentration (14 ± 0.5 g/dl) and haematocrit (42 ± 0.9) at SL and HA, suggesting similar hydration conditions.

**Figure 1:**
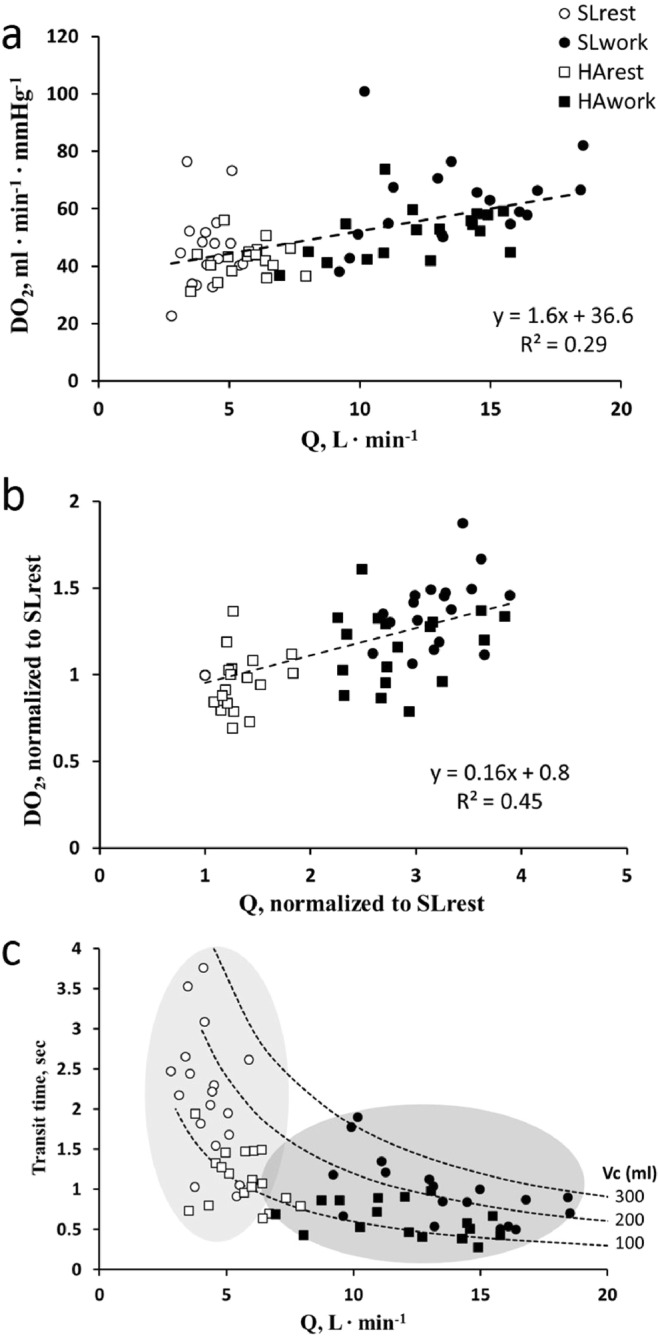
Correlation between diffusive and perfusive variables involved in O_2_ equilibration. (**a**) Correlation between O_2_ diffusive capacity (*DO*_*2*_) and cardiac output ($\dot{Q}$) for all subjects at SLrest (open circles), SLwork (closed circles), HArest (open squares) and HAwork (closed squares). (**b**) Correlation between *DO*_*2*_ and $\dot{Q}$, normalized to values at sea level rest (SLrest). (**c**) Correlation between *Tt* and $\dot{Q}$: figure also reports three calculated Iso-*Vc* dotted lines as labelled. Light grey and dark grey areas encompass rest and work data respectively.

Figure 1a shows the overall relationship between *DO*_*2*_ and cardiac output in the four conditions considered as indicated. Figure 1b shows the changes in *DO*_*2*_ and $\dot{Q}$ relative to SLrest that allows to appreciate that the relative increase in $\dot{Q}$ is greater than that of *DO*_*2*_. Figure 1c shows the distribution of *Tt vs* $\dot{Q}$ in the four conditions; the figure also reports the calculated iso-*Vc* lines as indicated. The light grey area, encompassing the open points referring to resting condition, suggests that exposure to HA leads to a decrease in *Tt* essentially related to a decrease in *Vc*. Conversely, the dark grey area referring to working conditions both at SL and HA suggests that the decrease in *Tt* mostly relates to the increase in $\dot{Q}$.

### Time-dependence of alveolo-capillary equilibration

**Figure 2:**
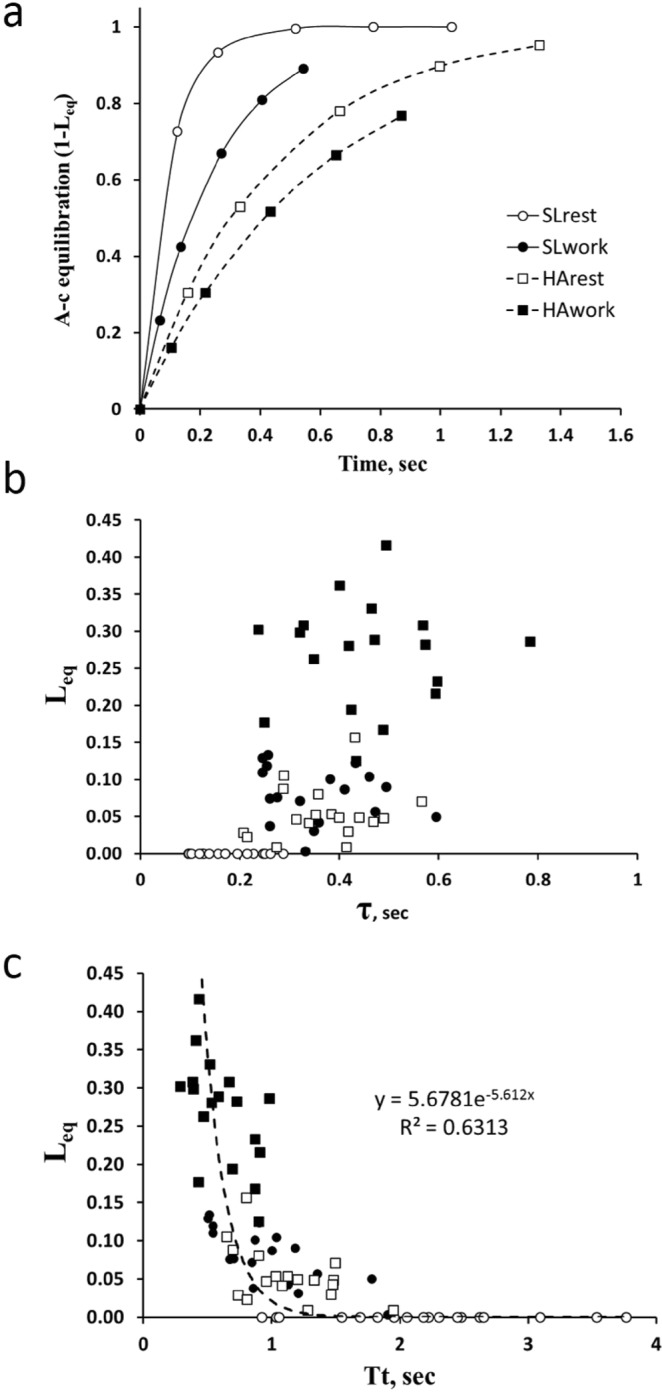
Time-dependence of alveolo-capillary equilibration. (**a**) Profile of alveolo-capillary (**A**–**c**) equilibration as a function of time along the pulmonary capillary for one subject at SLrest (open circles), SLwork (closed circles), HArest (open squares) and HAwork (closed squares); *see Appendix* for the definition of 1-L_eq_. (**b**) Plot of *L*_*eq*_ vs time constant (*τ*) for all subjects at SLrest, SLwork, HArest and HAwork. (**c**) Correlation between *L*_*eq*_ vs transit time (*Tt*) in all conditions.

In Fig. 2a we plotted for one subject the time course of the equilibration process to reach the final *L*_*eq*_ value attained at the exit of the pulmonary capillary in the four conditions (as indicated by symbols): the rightward displacement of the curves reflects the progressive increase in *τ* shifting from SLrest to SLwork, HArest and HAwork. The final value of *L*_*eq*_ clearly results from the matching of *τ* and *Tt*. Figure 2b shows the wide spectrum of the increase in *L*_*eq*_ when plotted vs the increase in *τ*. A stronger correlation was found between *L*_*eq*_ and *Tt* (Fig. 2c).

### Dependence of Leq from O_2_ diffusion and transport

**Figure 3:**
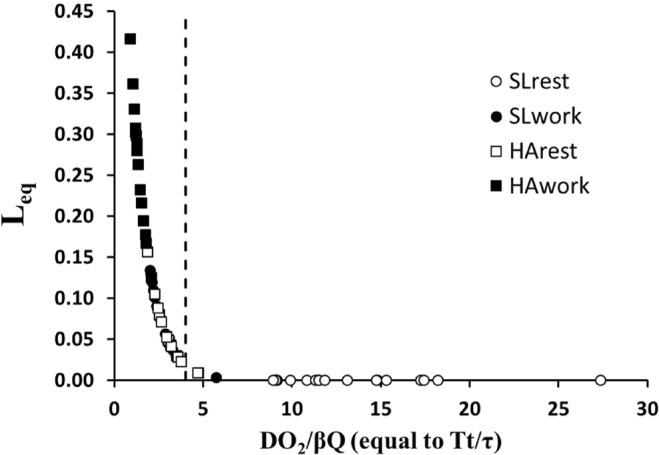
Dependence of *Leq* from O_2_ diffusion and transport. Relationship between diffusion equilibration (*L*_*eq*_) and *DO*_*2*_/*β*$\dot{Q}$ (that is equal to *Tt/τ*) at SLrest (open circles), SLwork (closed circles), HArest (open squares) and HAwork (closed squares). The vertical dashed line for *DO*_*2*_/*β*$\dot{Q}$ = 4 is a cutoff value below which a remarkable increase in *L*_*eq*_ occurs.

Considering the *β* values for the four conditions, we calculated from Eq. 3 the *L*_*eq*_ values that were plotted in Fig. 3 *vs DO*_*2*_/*β*$\dot{Q}$ (that is equal to *Tt*/*τ* according to equation A.6). Clearly, the relationship shows that a remarkable increase in *L*_*eq*_ occurs shifting from SL to HA when the value of abscissa drops below 3–4 (vertical dashed line).

**Figure 4:**
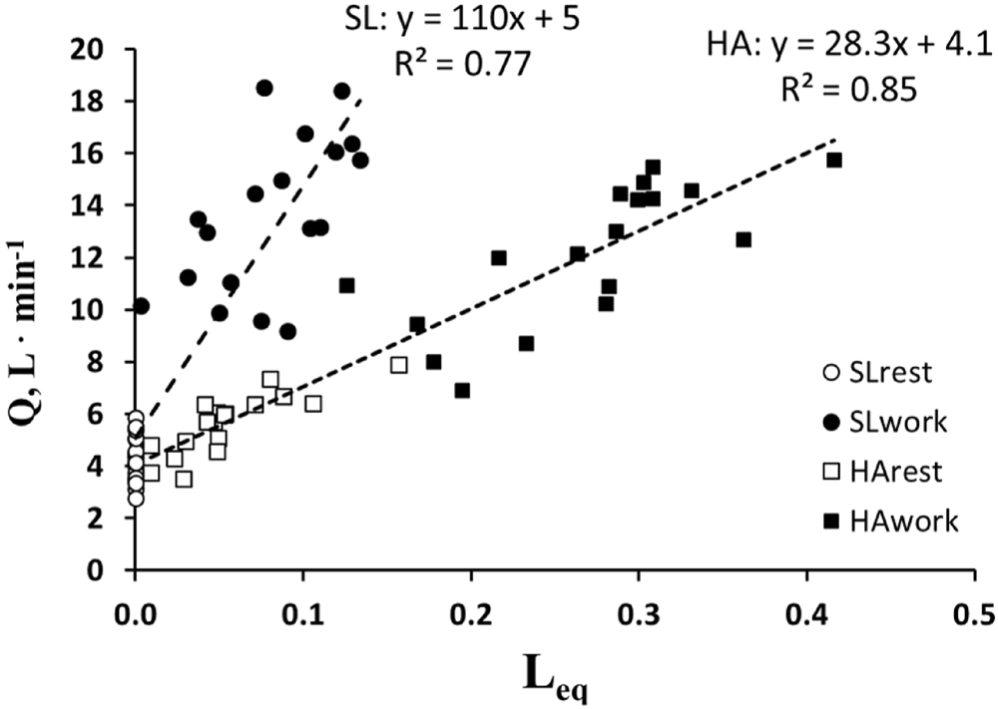
Correlation between *Leq* and cardiac output. Relationships between cardiac output ($\dot{Q}$) *vs* diffusion equilibration (*L*_*eq*_) for SL (rest + work) and for HA (rest + work).

Figure 4 shows that the cardiac output increases with increasing *L*_*eq*_. Note that data referring to HA lay on a relationship that is displaced to the right.

## Discussion

In general, O_2_ equilibration is considered to reflect the heterogeneity of regional distribution of ${\dot{V}}_{A}/\dot{Q}$ ratio^17–19^ as well as regional distribution of the diffusion/perfusion ratio^20^, both remaining difficult to be assessed. The model of alveolo-capillary equilibration that we considered circumvents this complication, and is open to different functional readings. An invasive approach, requiring an arterial and venous blood draw, would allow to define the ratio $\frac{{P}_{A}-{P}_{a}}{{P}_{A}-{P}_{\bar{v}}}$ (equation A.2). Conversely, a non-invasive approach would allow to estimate *DO*_*2*_/*β*$\dot{Q}$ that highlights the ratio between O_2_ diffusion capacity to O_2_ transport capacity; further, this approach allows to provide a time-based interpretation of the alveolo-capillary equilibration process based on *τ* and *Tt*.

As from Table 1, the average values of %*SatO*_*2*_ and *P*_*A*_ are in line for a group of healthy subjects and do not show remarkable variability. Yet, the individual values of *L*_*eq*_ varied subtantially reaching a value of 0.42 in the most demanding oxygen condition. This reveals considerable inter-individual differences concerning the efficiency of the O_2_ diffusion-transport system, that can be detected by the present approach.

The equilibrium reached at the exit of the pulmonary capillary results from a complex interaction of the functional parameters involved in the adaptive response to match O_2_ uptake with metabolic needs. The increase in *DO*_*2*_ favors the increase in O_2_ uptake and furthermore the increase in cardiac output favors oxygen delivery to the periphery (Fig. 1a): yet, in general, cardiac output ($\dot{Q}$) increased more than *DO*_*2*_ (Fig. 1b), so that the ratio *DO*_*2*_/$\dot{Q}$ inevitably decreased. Figure 1c allows to focus on some functional relationship between the three key parameters analyzed, namely *Tt*, $\,\dot{Q}$ and *Vc* when facing an increase in metabolic O_2_ requirement. The suggestion is that, on exposure to HA at rest (light grey area), the factor mostly affecting the lack of equilibration is the decrease in *Tt* due to a decrease in *Vc*, reflecting in turn pulmonary vasoconstriction^3^. Considering a cutoff of *Vc* = 200 ml, one can note that during work at SL (Fig. 1c, closed circles) 72% of the subjects had a *Vc* > 200 ml, while at SLrest (Fig. 1c, open circles) they amounted only to 22%: accordingly, SLwork led, on the average, to an increase in *Vc*. Despite some inter-individual variability, the increase in *Vc* appears useful to limit a decrease in *Tt* (levelling, on the average, to ~0.75 sec) due to the remarkable increase in $\dot{Q}$. Considering now HAwork (closed squares), it appears that *Tt* and corresponding *Vc* remain in the lower range of measured values.

It is noteworthy in this respect that pulmonary capillary vasomotion in hypoxia, as deduced from changes in *Vc*, was found to be variable among individuals^3^, being interpreted as vasoconstriction or vasodilation. Recent data on *in vivo* imaging of alveolar units in an animal experimental model of hypoxia exposure clearly indicate heterogeneous alveolar blood flow distribution, with a more pronounced vasoactive response in larger alveolar units showing signs of interstitial edema^21^. Vasoconstriction may in fact be regarded as the main mechanism aimed at preventing the aggravation of developing edema, an interpretation pivoting around the perturbation induced by hypoxia on the capillary interstitial fluid dynamics in the lung^22,23^.

The role of *Tt* has been considered either as a non limiting^24^ or a potentially limiting^25,26^ factor to blood oxygenation. As Fig. 2a suggests, the alveolo-capillary O_2_ equilibration depends from the relative changes of both *τ* and *Tt*. Figure 2b shows a great dispersion by plotting the increase in *L*_*eq*_ with increase in *τ*, reflecting the variability of the individual ratio *Vc/DO*_*2*_ (equation A.5). Yet, a more significant correlation was found by plotting *L*_*eq*_ vs *Tt* (Fig. 2c). The increase in *L*_*eq*_ obviously reflects the decrease of the *DO*_*2*_/$\dot{Q}$ and of *Tt/τ* ratio (Fig. 3): this figure, although representing a “truism”, wishes to emphasize the remarkable increase in *L*_*eq*_ when *Tt*/*τ* drops below ~4 (dashed vertical line).

It is noteworthy that, both at SL and HA, the highest cardiac output increased with increasing individual *L*_*eq*_ (Fig. 4) revealing the need for a greater O_2_ transport to the tissues in face of increasing lack of O_2_ equilibration. Further, it is noteworthy that such response is highly variable among individuals. Bradycardia at SLrest favors O_2_ equilibration on increasing oxygen metabolic need to buffer the decrease in *Tt/τ*.

## Comparison with Previous Models of Oxygen Uptake and Transport

The quantitative interaction between diffusive and convective factors along the oxygen transfer pathway led to the development of functional models aiming to identify factors causing a potential limitation to the maximum oxygen consumption ($\dot{V}{O}_{2max}$). The present analysis is not primarily considering the point of $\dot{V}{O}_{2max}$, that includes the oxygen transport from the lungs to the mitochondria; it only considers the diffusive/convective function at alveolar level in subthreshold exercises. It appears therefore reasonable to compare the present results with predictions from two relevant models of oxygen transport, referred here as Wagner’s and di Prampero’s models, as reviewed by Ferretti^27^. Wagner’s model^28^ described oxygen transport by derivation of Roughton and Forster^8^ equation (Eq. 1) to express O_2_ uptake as a function of membrane diffusion for O_2_ and capillary blood volume *Vc*, ignoring the alveolar-capillary coupled diffusion/transport for O_2_. Just considering this last point, we are able to estimate inter-individual differences of transit time (*Tt*) and time constant (*τ*) of the alveolo-capillary O_2_ equilibration. Our data, compared to predictions from Wagner’s model, indicate transit times about 3 times longer and equilibration kinetics remarkably slowed down. To specifically deal with hypoxia, Wagner’s model evolved further^29^ by setting a 3-equations algebraic system to include the role of ventilation, of the alveolar diffusive/transport^5^ and of O_2_ diffusion at the periphery. The model goes on to speculate on maximum exercise at top of Mount Everest, 8848 m. Data fed into the algebraic system were: lung ventilation of 150 L · min^−1^, *DO*_*2*_ of 160 ml · min^−1^ · Torr^−1^ and cardiac output $\dot{Q}$ of 18 L · min^−1^. A comment concerning these assumptions is due. The limit to the increase in maximum ventilation beyond which any increase in ventilation does not result in O_2_ available for useful external work, was set, based on respiratory mechanics studies^30^ and energy cost as well as efficiency of breathing^31^ at about 120 L · min^−1^. Furthermore, actual measurements done at high altitude confirmed that the energy cost of extra ventilation outstripped the interest for increasing oxygen transfer^32^. The assumption of *DO*_*2*_ = 160 ml · min^−1^ · Torr^−1^ appears exceedingly high and moreover, the decrease in cardiac output at Everest summit was assumed at 23% relative to sea level value of 25 L · min^−1^, rather than an expected 40%^33^. Based on the assumptions^29^, one could estimate *L*_*eq*_ ~0.5 at summit of Everest, a rather low value being essentially equal to that corresponding to sub-threshold workload at ~4000 m (Fig. 4, HA), a much lower altitude. Nevertheless, there is agreement with Wagner’s model that in normoxia a limitation of the alveolo/capillary function is essentially low, while in severe hypoxia, the diffusion limitation may account for 80% of the alveolar-capillary *PO*_*2*_ difference^19^. Di Prampero’s model^34,35^, based on an electrical analogue, considers O_2_ flow to occur down a system of resistances placed in series. The finding of low Leq values in normoxia confirms the conclusion from di Prampero’s model that ventilation has a minimal role to limit $\dot{V}{O}_{2max}$, due to the flatness of the Hb-O_2_ relationship at normoxic *PO*_*2*_^35^. Di Prampero and Ferretti^35^ introduced the concept of non-linear behaviour of the respiratory system in hypoxia (P_I_O_2_ ~ 90 Torr) when shifting to the vertical part of the Hb-O_2_ dissociation curve^35^. Our model implies mathematically the non linearity of O_2_ transport at the alveolar-capillary level when shifting from normoxia to hypoxia, as shown by the exponential increase in *L*_*eq*_ (see Fig. 3): this occurs because the increase in O_2_ diffusion, due to the greater binding capacity of the blood, is less than the increase in cardiac output. As shown in Fig. 4, this discrepancy becomes larger with increasing the metabolic requirement. Finally, our conclusions agree with those from Di Prampero and Ferretti^35^ that in hypoxia the limitation imposed by diffusion exceeds that of the cardiovascular transport.

## Limitations of the Study

- The same *β* values were used for all subjects according to the condition considered. At SL inter-individual differences in slope (tangent of the function) may well exist, although they must remain on mathematical ground within narrow limits due to the low slope of the Hb-dissociation curve; at HA below *PO*_*2*_ of 55 mmHg the slope of Hb-dissociation curve is obviously higher than at SL but fairly constant.
- The estimate of cardiac output in exercise, relative to supine, based on a fixed 40% increase for stroke volume multiplied by heart rate introduces a noise into the analysis, whose degree cannot be quantitated.
- The present analysis allows to quantitate the inter-individual differences in alveolo-capillary oxygen equilibration but is unable to quantitate the relative contribution of the factors leading to such differences, namely: the heterogeneity in ventilation/perfusion ratio, the shunt effect as well as the actual values of *P*_*a*_ and ${P}_{\bar{v}}$.

## Data Availability

Authors declare no restrictions on the availability of materials or information at the time of submission from the corresponding author on reasonable request.

## Appendix

The oxygen alveolo-capillary equilibration at the exit of the pulmonaty capillary has been described as^5^:

A1 $${P}_{A}-{P}_{a}=({P}_{A}-{P}_{\bar{v}})\,{e}^{-\frac{D{O}_{2}}{\beta \dot{Q}}}$$

where, *P*_*A*_, *P*_*a*_ and ${P}_{\bar{v}}$ are the partial pressures of O_2_ in the alveoli, in the arterial blood leaving the lung and in the mixed venous blood, respectively; *DO*_*2*_ is the oxygen diffusive capacity, $\dot{Q}$ is the cardiac output and *β* is the hemoglobin binding capacity for oxygen, expressing the mean slope of the Hb-O_2_ dissociation curve. This approach is grounded on the equality between O_2_ diffusion and O_2_ transport under steady state condition.

The conceptual development of Eq. (2) reported in Methods allows to define the potential limitation of the O_2_ equilibration at the exit of pulmonary capillary as:

A2 $${L}_{eq}=\frac{{P}_{A}-{P}_{a}}{{P}_{A}-{P}_{\bar{v}}}={e}^{-\frac{D{O}_{2}}{\beta \dot{Q}}}$$

*L*_*eq*_ can vary from 0 (the case of perfect equilibration) to 1 (the case of shunt).

Knowing *Vc* and $\dot{Q}$, one can derive the average pulmonary blood capillary transit time (*Tt*) as:

A3 $$Tt=\frac{Vc}{\dot{Q}}$$

One can therefore reformulate Eq. (2) reported in the Methods as:

A4 $${L}_{eq}={e}^{-\frac{D{O}_{2}}{\beta }\cdot \frac{Tt}{Vc}}$$

So that one can explicit the time constant *τ* of the exponential equilibration process as:

A5 $$\tau =\frac{\beta Vc}{D{O}_{2}}$$

Knowing *Tt* and *τ*, one can re- define *L*_*eq*_ at the exit from the pulmonary capillary as:

A6 $${L}_{eq}={e}^{-\frac{Tt}{\tau }}$$

Knowing the transit time *Tt*, allows us to describe the O_2_ equilibration process as a function of time. Revising Eq. (A.4), one has:

A7 $$Leq\,(t)={e}^{-\frac{D{O}_{2}}{\beta \dot{Q}}\cdot \frac{t}{{T}_{t}}}$$

for time (*t*), being a fraction of known transit time (*Tt*).

For the sake of graphical representation of the alveolo-capillary gradient (*see* Fig. 2a), we put on the ordinate alveolo-capillary (A-c) equilibration as 1-*L*_*eq*_, meaning that in case of perfect equilibration one has A-c equilibration equal to 1, for *L*_*eq*_ = 0.

*Estimate of β*. We relied on analytical treatment of the hemoglobin dissociation curve^36^. Average slopes of the curve were estimated considering *PCO*_*2*_ values at SL and HA. For the range of *PO*_*2*_ considered, we derived *β* values for SLrest, SLwork, HArest and HAwork as follows: 0.83, 1.72, 2.5 and 3.25 ml · L^−1^ · mmHg^−1^, respectively.
